# Antibiotic treatment recommendations for acute respiratory tract infections in Scandinavian general practices—time for harmonization?

**DOI:** 10.1080/02813432.2024.2422441

**Published:** 2024-11-04

**Authors:** Malene Plejdrup Hansen, Sigurd Høye, Katarina Hedin

**Affiliations:** a Center for General Practice, Aalborg University, Aalborg, Denmark; b Research unit for General Practice, Institute of Public Health, University of Southern Denmark, Odense, Denmark; c Department of General Practice, Institute of Health and Society, Antibiotic Centre for Primary Care, University of Oslo, Oslo, Norway; d Futurum, Region Jönköping County, Jönköping, Sweden; e Department of Health, Medicine and Caring Sciences, Linköping University, Sweden; f Department of Clinical Sciences in Malmö, Family Medicine, Lund University, Malmö, Sweden

**Keywords:** General practice, antibiotics, acute respiratory tract infections, guidelines, phenoxymethylpenicillin

## Abstract

**Introduction:**

During recent years, the world—including Scandinavia—has experienced significant challenges with shortages of antibiotics. In Scandinavia, phenoxymethylpenicillin is recommended as first-line antibiotic treatment for most acute respiratory tract infections (ARTIs). However, the Scandinavian countries each constitute rather small markets for phenoxymethylpenicillin. The aim of this discussion paper is to enlighten the differences in Scandinavian ARTI antibiotic treatment recommendations. This information is fundamental for exploring the potential of harmonizing treatment recommendations in Denmark, Norway and Sweden—to help ensure sufficient future supply of phenoxymethylpenicillin.

**Methods:**

Information from national ARTI antibiotic treatment recommendations from respectively Denmark, Norway and Sweden has been collated.

**Results:**

Several discrepancies exist in recommendations. Adult dosage varies from a minimum of 660 mg x 4 (Denmark) to a maximum of 2000 mg × 3 (Sweden). Within Norway and Sweden, variations in recommended dosage also exist between the different types of ARTIs. A main challenge is that the tablet strengths recommended, and available on the market in the three countries, differs.

Also, antibiotic treatment durations vary significantly between countries and infections treated—from five to 10 days of treatment.

**Conclusion:**

In the capacity of a well-established network for antibiotic stewardship, we have enlightened the differences in Scandinavian ARTI antibiotic treatment recommendations. This paper is the first step moving forward to scrutinizing the potential for harmonizing recommendations for Denmark, Norway and Sweden—to help ensure continued supply of phenoxymethylpenicillin for use within the Scandinavian countries.

Most antibiotics are issued in general practice—with acute respiratory tract infections (ARTIs) being the main indications [1,2]. ARTI is an overall term used for infections related to the respiratory tract. Many of these infections, such as common cold and influenza, are caused by virus, and do not need antibiotic treatment. However, some ARTIs, such as acute otitis media, acute rhinosinusitis, acute pharyngo-tonsilitis and pneumonia, sometimes require antibiotic treatment.

In Scandinavia, phenoxymethylpenicillin is recommended as first-line antibiotic treatment for most ARTIs when antibiotics are warranted [3–5]. Phenoxymethylpenicillin is a narrow-spectrum antibiotic covering the most commonly causative ARTI bacterial pathogens like *Streptococcus pyogenes* and *Streptococcus pneumonia* [6]. The levels of penicillin resistance in pneumococci are significantly lower in Scandinavia than in many other European countries. This is probably due to our prudent use of antibiotics and is the basis for us recommending phenoxymethylpenicillin as the first choice. Therefore, there is good reason to strive to maintain the Scandinavian treatment tradition - and involve other countries that also wish to learn from us [7].

## Shortages of antibiotics

During recent years, the world—including Scandinavia—has experienced significant challenges with shortages of antibiotics [8]. The Scandinavian countries each constitute rather small markets. Phenoxymethylpenicillin is used only to a limited extent in countries outside Scandinavia [9]. This makes us particularly vulnerable. In Denmark, for example, shortage of phenoxymethylpenicillin 660 mg emerged—resulting in a markedly price increase [10]. Consequently, many GPs converted to treatment with less expensive phenoxymethylpenicillin 800 mg tablets. Also, in Norway and Sweden similar situations have caused prescribing of alternative dosages and pack sizes—and ultimately recommendations of using more broad-spectrum antibiotics [11,12]. Moreover, some phenoxymethylpenicillin tablets for children (165 and 330 mg) have been removed from the Norwegian marked due to low sales in the limited Norwegian market.

The European Commission, the Heads of Medicines Agencies, and the European Medicines Agency have recently issued recommendations for actions to be taken to avoid shortages of key antibiotics to treat ARTIs in the upcoming winter season [13]. One of the recommendations is ‘increase the production of key antibiotics’, with phenoxymethylpenicillin being one of the key antibiotics mentioned.

## Scandinavian ARTI antibiotic treatment recommendations

In the capacity of a well-established Scandinavian network for antibiotic stewardship, we decided to collate information from national ARTI antibiotic treatment recommendations with the aim of enlightening the differences in recommendations. This information is fundamental for exploring the potential of harmonizing treatment recommendations in Denmark, Norway and Sweden—to help ensure sufficient future supply of phenoxymethylpenicillin.

Recommendations for first-line antibiotic treatment of acute otitis media, acute rhinosinusitis, acute pharyngo-tonsilitis and community-acquired pneumonia are presented in Table 1.

**Table 1.** First-line antibiotic treatment of acute respiratory tract infections in general practice.

	Denmark [3]	Norway [5]	Sweden [4]
**Acute otitis media**	*Children*Phenoxymethylpenicillin 50 mg/kg × 3 for 5 days *Adults* [15] **Phenoxymethylpenicillin 660 mg* × 4 for 5 days	*Children*Phenoxymethylpenicillin 10 mg/kg × 4 for 5 days *Adults*Phenoxymethylpenicillin 1000 mg × 4 for 5 days	*Children* Phenoxymethylpenicillin 25 mg/kg × 3 for 5 days *Adults*Phenoxymethylpenicillin 1600 − 2000 mg × 3 for 5 days
**Acute rhinosinusitis**	*Children* [16] Phenoxymethylpenicillin 50 mg/kg/day × 3 for 7 days *Adults*Phenoxymethylpenicillin 660 mg* × 4 for 5 days	*Children*Phenoxymethylpenicillin 15 mg/kg × 4 for 7 days *Adults*Phenoxymethylpenicillin 1000 mg × 4 for 7 days	*Children* Phenoxymethylpenicillin 25 mg/kg × 3 for 7 days *Adults*Phenoxymethylpenicillin 1600 − 2000 mg × 3 for 7 days
**Acute pharyngo-tonsillitis**	*Children*Phenoxymethylpenicillin 50 mg/kg × 3 for 5 days *Adults*Phenoxymethylpenicillin 660 mg* × 4 for 5 days	*Children*Phenoxymethylpenicillin 10 mg/kg × 4 for 5 days *Adults*Phenoxymethylpenicillin 660 mg × 4 for 5 days	*Children*Phenoxymethylpenicillin 12,5 mg/kg × 3 for 10 days*Adults*Phenoxymethylpenicillin 1000 mg × 3 for 10 days
**Community-acquired pneumonia**	*Children*Phenoxymethylpenicillin 50 mg/kg × 3 for 5 days *Adults*Phenoxymethylpenicillin 660 mg* × 4 for 5 days	*Children*Phenoxymethylpenicillin 15 mg/kg × 4 for 7 days *Adults*Phenoxymethylpenicillin 1000 mg × 4 for 7 days	*Children*Phenoxymethylpenicillin 12,5 mg/kg × 3 for 7 days *or* Amoxicillin 15mg/kg × 3 for 5 days*Adults*Phenoxymethylpenicillin 1000 mg × 3 for 7 days

*Phenoxymethylpenicillin 800 mg is recommended as equal choice of treatment to 660 mg.

Several discrepancies exist, for example, adult dosage varies from a minimum of 660 mg × 4 (Denmark) to a maximum of 2000 mg × 3 (Sweden). Within Norway and Sweden, variations in recommended dosage also exist between the different types of ARTIs. Also, antibiotic treatment durations vary significantly between countries and infections treated—from five to 10 days of treatment.

## Variation in phenoxymethylpenicillin tablet strengths

As illustrated above, recommendations for phenoxymethylpenicillin treatment of ARTIs vary significantly between the three countries. Importantly, not only recommended dosage and treatment duration differ - but also the strengths of phenoxymethylpenicillin tablets available within each of the countries vary largely. In Denmark, phenoxymethylpenicillin is available in tablets of either 660 mg and 800 mg, while in Norway 650/660 mg and 1000 mg are accessible, and in Sweden tablets of 250, 500, 800 and 1000 mg are sold.

A recently published report from the collaboration platform PLATINEA (PLATform för Innovation av Existierende Antibiotika), which aims to improve the availability and use of antibiotics, shows that phenoxymethylpenicillin tablets are marketed in a total of 10 strengths and in 41 different package sizes in Denmark, Finland, Norway and Sweden [14]. The authors of the report argue that less fragmentation will provide better availability.

Collectively, the Scandinavian countries constitute a fairly large market, equivalent to 22 million inhabitants. However, a main challenge is that the tablet strengths recommended, and available on the market in our three countries, differs. Consequently, for example, Norwegian guidelines cannot be followed in Sweden and Denmark and vice versa.

## Harmonizing recommendations?

In both Denmark, Norway and Sweden, recommendations for antibiotic treatment of ARTIs are mainly developed by general practitioners/researchers within the field of general practice. However, also close collaborations with other specialists, for example, specialists in clinical microbiology, otorhinolaryngology and respiratory medicine, influence the content of national recommendations.

Since the Scandinavian populations are alike, and the resistance patterns in the most common ARTI bacteria are about the same it would be valuable to thoroughly investigate the potential of aligning treatment recommendations within the three countries. Harmonizing recommendations for Denmark, Norway, and Sweden has the potential of assisting in a sufficient continued supply of phenoxymethylpenicillin for use within the Scandinavian countries. However, several other mechanisms, such as actions taken by Medicines Agencies or drug companies, also influence access to phenoxymethylpenicillin in Scandinavia. Ultimately, a joint action needs to be taken by both clinicians, researchers, drug companies and Health Authorities to ensure a sufficient continued supply of phenoxymethylpenicillin for use within the Scandinavian countries.
